# Single molecule analysis of effects of non-canonical guide RNAs and specificity-enhancing mutations on Cas9-induced DNA unwinding

**DOI:** 10.1093/nar/gkz1058

**Published:** 2019-11-12

**Authors:** Ikenna C Okafor, Digvijay Singh, Yanbo Wang, Minhee Jung, Haobo Wang, John Mallon, Scott Bailey, Jungjoon K Lee, Taekjip Ha

**Affiliations:** 1 Department of Biology, Johns Hopkins University, Baltimore, MD 21218, USA; 2 Department of Biophysics and Biophysical Chemistry, Johns Hopkins University School of Medicine, Baltimore, MD 21205, USA; 3 Toolgen, Seoul 08501, Republic of Korea; 4 Bloomberg School of Public Health, Johns Hopkins University School of Medicine, Baltimore, MD 21205, USA; 5 Department of Biophysics, Johns Hopkins University, Baltimore, MD 21218, USA; 6 Department of Biomedical Engineering, Johns Hopkins University, Baltimore, MD 21205, USA; 7 Howard Hughes Medical Institute, Baltimore, MD 21205, USA

## Abstract

Cas9 has made a wide range of genomic manipulation possible. However, its specificity continues to be a challenge. Non-canonical gRNAs and new engineered variants of Cas9 have been developed to improve specificity, but at the cost of the on-target activity. DNA unwinding is a checkpoint before cleavage by Cas9, and was shown to be made more sensitive to sequence mismatches by specificity-enhancing mutations in engineered Cas9s. Here we performed single-molecule FRET-based DNA unwinding experiments using various combinations of non-canonical gRNAs and different Cas9s. All engineered Cas9s were less promiscuous than wild type when canonical gRNA was used, but HypaCas9 had much-reduced on-target unwinding. Cas9-HF1 and eCas9 showed the best balance between low promiscuity and high on-target activity with canonical gRNA. When extended gRNAs with one or two non-matching guanines added to the 5′ end were used, Sniper1-Cas9 showed the lowest promiscuity while maintaining high on-target activity. Truncated gRNA generally reduced unwinding and adding a non-matching guanine to the 5′ end of gRNA influenced unwinding in a sequence-context dependent manner. Our results are consistent with cell-based cleavage data and provide a mechanistic understanding of how various Cas9/gRNA combinations perform in genome engineering.

## INTRODUCTION

CRISPR enzymes complexed with programmable guide-RNA (gRNA) can target complementary sequences in DNA or RNA, and their applications have revolutionized biology (1,2). One of the most widely used CRISPR enzymes is Cas9, and the most well studied Cas9 is from *Streptococcus pyogenes* (SpCas9). SpCas9 in complex with a gRNA (Cas9–RNA) binds 20 bp long complementary DNA sequences known as the protospacer, which follows an essential binding region on the DNA called the protospacer adjacent motif (PAM). After stable binding of Cas9 to the PAM sequence, there is directional unwinding of the protospacer and concomitant hybridization between gRNA and protospacer target strand to form Cas9–RNA–DNA (3–8,25). Following unwinding, Cas9–RNA activates its two nuclease domains (HNH for target strand and RuvC for non-target strand) and cleaves the DNA, producing a double-strand break (3–9,40).

**Figure 1.**
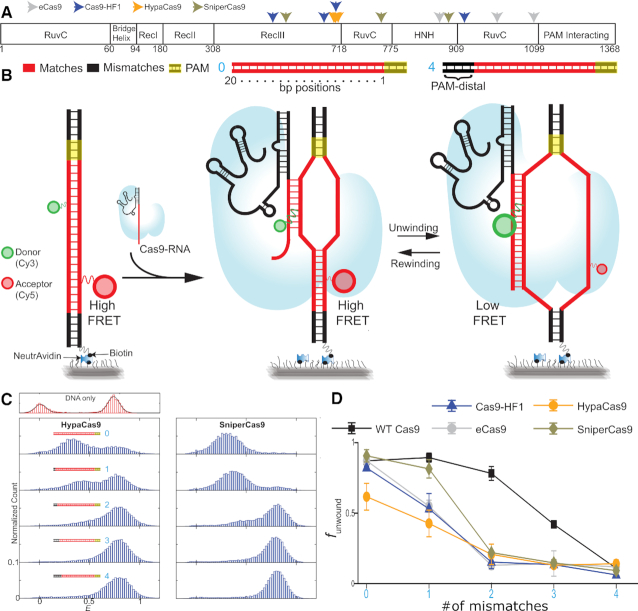
DNA unwinding by HypaCas9 and SniperCas9. (**A**) Schematic of the domain organization of Cas9. The positions of mutations belonging to different EngCas9 are indicated. (**B**) Schematic of a smFRET assay for investigation of Cas9-RNA induced DNA unwinding of surface immobilized DNA incubated against free Cas9-RNA in solution. Cognate DNA and DNA with mismatches (no complementarity) against the gRNA at the indicated PAM-distal site in the protospacer region were used in this assay. The number of PAM-distal (*n*_PD_) mismatches is shown in cyan digits. (**C**) *E* histograms for DNA with different *n*_PD_ = 0, 1, 2, 3 and 4 (cyan digits) for dHypaCas9 and dSniperCas9. (**D**) *f*_unwound_ versus *n*_PD_ for different Cas9s. Data for dCas9, deCas9 and dCas9-HF1 is taken from a previous study (31). Error bars represent standard deviation (SD).

Several derivatives of SpCas9, here referred to as wild type or WT Cas9, have been engineered (EngCas9s) to improve cleavage specificity (10–17,19) (Figure 1A). Enhanced Cas9 (eCas9) and High Fidelity (Cas9–HF1) were generated by rationally introducing mutations in various domains, which resulted in a higher threshold for nuclease activation. We previously showed that the mutations make DNA unwinding more sensitive to mismatches and slow down cleavage from the unwound state (18). In Hyper accurate Cas9 (HypaCas9), two mutations from Cas9 HF1 was restored to wild type, improving on-target activity. Sniper1–Cas9 (SniperCas9) was developed using a directed evolution approach, and its on-target activity was similar to the wild type in mammalian cells.

We previously showed that Cas9s bind DNA ultrastably (lifetime > 1 h) if there are 9–10 bp complementarity between the PAM-proximal end of the protospacer and the gRNA, but cleavage requires higher complementarity (∼16 bp for WT Cas9 and ∼17–18 bp for EngCas9s) (5,7,18). Because even the specificity-enhancing variants of Cas9s still tolerate mismatches between gRNA and protospacer with potentially undesirable side effects, additional strategies such as the use of truncated (20) and extended gRNAs (21,22), collectively referred to as non-canonical gRNAs, have been employed and shown to improve genome editing efficiency or specificity in some cases. However, the mechanisms underlying their improvements remain poorly understood. Here, we have employed an smFRET DNA unwinding assay (Figure 1B) to investigate the effects of pairing non-canonical gRNAs (20–22) with EngCas9s on DNA unwinding (Supplementary Figure S1). Our results are consistent with cell-based cleavage data and provide a mechanistic understanding of why certain Cas9/gRNA combinations perform better or worse in genome engineering which may also guide future applications and further improvements.

## MATERIALS AND METHODS

### Preparation of DNA targets

The schematic of the DNA target used in the smFRET assay to investigate Cas9–RNA induced DNA unwinding is shown in Supplementary Figures S2A and S3. Integrated DNA Technologies (IDT, Coralville, IA, USA) was the commercial supplier of all DNA oligonucleotides. For introducing Cy3 and Cy5 labels at the indicated locations, the oligonucleotides were purchased with amine-containing modified thymine at the indicated location. A C6 linker (amino-dT) was used to label the DNA strands with Cy3 or Cy5 *N*-hydroxysuccinimido (NHS). For preparing the DNA, the constituents non-target strand, target strand, and a 22 nt biotinylated adaptor strand were first mixed in 10 mM Tris–HCl, pH 8 and 50 mM NaCl. The mixture was then transferred to a heat block preheated to 90°C. After 2 min of heating, the mixture was taken off the heat block and allowed to cool to room temperature over a few hours. The sequences of the target and non-target strand (with same label positions) were changed to create DNA targets with mismatches. The full sequence of all DNA targets used in the smFRET assay is shown in Supplementary Table S1.

### Expression and purification of Cas9

The protocols for Cas9 expression and purification have been described previously (3,5,18). pET-based expression vectors were used for expression of all Cas9s except SniperCas9 and dSniperCas9. For WT Cas9, the vector consisted of sequence encoding Cas9 (1–1368 residues of Cas9 from *Streptococcus pyogenes*) and an N-terminal decahistidine-maltose binding protein (His10-MBP) tag, followed by a peptide sequence containing a tobacco etch virus (TEV) protease cleavage site. This vector was used as a base vector for other Cas9s. The mutations for catalytically inactive cas9 (dCas9) and/or EngCas9 mutations were introduced by site-directed mutagenesis kits (QuickChange Lightning; Agilent Technologies, Santa Clara, CA, USA). Around-the-horn PCR was performed on the base vector for creating HypaCas9 mutations.

The cells were grown in TB (Terrific Broth) or 2YT medium (higher expression obtained for TB) at 37°C for a few hours and the cell density was continuously monitored by measuring the optical density of the media at 600 nm (OD_600_). Cas9 expression was induced when the OD_600_ reached 0.6 with 0.5 mM IPTG, and the temperature was lowered to 18°C. The cells were induced for 12–16 h. Following which, the media containing cells were centrifuged to harvest the cells. The supernatant media, devoid of cells, was discarded. The harvested cells were then collected and lysed in the buffered solution containing 50 mM Tris pH 7.5, 500 mM NaCl, 5% glycerol, 1 mM TCEP, protease inhibitor cocktail (Roche) and with/without Lysozyme (Sigma Aldrich). Fisher Model 500 Sonic Dismembrator (Thermo Fisher Scientific) was used to homogenize the lysed cells by operating it at 30% amplitude in three 1-minute cycles, each consisting of series of 2 s sonicate–2 s repetitions, or the lysed cells were homogenized in a microfluidizer (Avestin). The solution was subjected to ultra-centrifugation at 15 000 g for 30–45 min to remove the cellular debris from the lysed and homogenized solution. Following which, the supernatant of lysate was collected, and cellular debris was discarded.

The supernatant was added to Ni-NTA agarose resin (Qiagen), and His10-MBP-TEV-Cas9 was allowed to bind Ni-NTA. The Ni-NTA bound with His10-MBP-TEV-Cas9 was then washed extensively with 50 mM Tris pH 7.5, 500 mM NaCl, 10 mM imidazole, 5% glycerol, 1 mM TCEP. The His10-MBP-TEV-Cas9 bound to Ni-NTA was then eluted in a single-step with the elution buffer of 50 mM Tris pH 7.5, 500 mM NaCl, 300 mM imidazole, 5% glycerol, 1 mM TCEP. For buffer exchange and to remove imidazole from the eluted solution containing Cas9, the eluted solution was dialyzed into 20 mM Tris–Cl pH 7.5, 125 mM KCl, 5% glycerol, 1 mM TCEP) overnight at 4°C. Concomitant with this dialysis, the cleavage of TEV-protease site by TEV protease was simultaneously carried out which resulted in free 10-His-MBP and Cas9 constituents in the solution. To remove and arrest away 10-His-MBP from the solution, the solution containing free 10-His-MBP and Cas9 constituents was subjected to another round of Ni-NTA agarose column purification resulting in the solution containing only Cas9. For further purification, this solution was subjected to size-exclusion chromatography on a Superdex 200 16/60 column (GE Healthcare) in Cas9 storage buffer (20 mM Tris–Cl pH 7.5, 100 mM KCl, 5 % glycerol and 5 mM MgCl_2_).

In some preparations, TEV cleavage and dialysis were not concomitant. The eluted solution containing His10-MBP-TEV-Cas9 fusion was first subjected to TEV protease treatment over a few hours. After TEV cleavage, the solution was then dialyzed into 20 mM Tris–Cl pH 7.5, 125 mM KCl, 5% glycerol, 1 mM TCEP for 3 h. The dialyzed solution was then applied to HiTrap SP HP sepharose column (GE Healthcare) and washed with 20 mM Tris–Cl pH 7.5, 125 mM KCl, 5% glycerol, 1 mM TCEP for three column volumes. The Cas9 bound to the column was then eluted into 20 mM Tris–Cl pH 7.5, 1 M KCl, 5% glycerol, 1 mM TCEP using a linear gradient from 0 to 100% over 20 column volumes. The eluted Cas9 was then further purified into Cas9 Storage Buffer (20 mM Tris–Cl pH 7.5, 200 mM KCl, 5% glycerol, 1 mM TCEP) by size exclusion chromatography on a Superdex 200 16/60 column (GE Healthcare). All the purification steps described in this section were performed at 4°C, and the purified Cas9 was stored at −80°C for long-term storage.

The SniperCas9 was also expressed and purified from *Escherichia coli*. The DNA sequence encoding SniperCas9 along with an NLS, HA epitope and His-tag at the N-terminus was cloned into pET28-b(+) vector. Using this vector, the recombinant protein was then expressed in *E. coli*, and first purified from the harvested cell lysate using Ni-NTA agarose beads (Qiagen). For buffer exchange, this purified protein was then dialyzed against 20 mM HEPES pH 7.5, 150 mM KCl, 1 mM DTT, and 10% glycerol. The Ultracel 100 K cellulose column (Millipore) was then used to concentrate the purified/dialyzed SniperCas9. The SDS-PAGE was used to analyze the concentration and purity of the final SniperCas9 protein.

### Preparation of gRNA and Cas9–gRNA

The canonical gRNA of Cas9 consists of CRISPR RNA (crRNA) and trans-activating crRNA (tracrRNA) (Supplementary Figure S2A) where the gRNA sequence hybridizing with the target strand of DNA target is 20 bp long (3,4). All gRNAs were prepared by mixing crRNA and tracrRNA in 1:1.2 ratio in 10 mM Tris HCl (pH 8) and 50 mM NaCl. This mixture was then placed in a heating block pre-heated to 90°C for 2 min. Following which, the mixture was allowed to cool to room temperature over a few hours for efficient hybridization between crRNA and tracrRNA. The non-canonical gRNA, i.e. truncated and extended gRNA was created by using the truncated and extended crRNA, respectively with the same tracrRNA (Supplementary Figure S3). Both the crRNA and tracrRNA were in-vitro transcribed as described previously and were purified to remove rNTPS using the commercial kits (Zymo Research). The in-vitro transcription produces RNA with 1–3 nt truncations and elongations at the 3′ end. Such likely truncations and elongations did not affect our experiments as the 3′ end of RNA have no role in Cas9-RNA activity, which was also confirmed by the control unwinding experiments using gel-purified fixed length RNAs which produced the same results as the kit-purified RNAs.Cas9-RNA was prepared by mixing the gRNA and Cas9 at a ratio of 1:3 in Cas9–RNA activity buffer of 20 mM Tris–HCl (pH 8), 100 mM KCl, 5 mM MgCl_2_, 5% (v/v) glycerol. The full sequences of all the gRNA used in this study are available in Supplementary Table S1.

### Single-molecule fluorescence imaging and data analysis

Flow chamber surfaces coated with polyethylene glycol (PEG) was used for immobilization of DNA targets. These flow chambers were purchased from Johns Hopkins University Microscope Supplies Core. The neutrAvidin–biotin interaction was used for immobilizing the biotinylated DNA target molecules on the PEG-passivated flow chamber in the Cas9–RNA activity and imaging buffer (20 mM Tris–HCl, 100 mM KCl, 5 mM MgCl_2_, 5% (v/v) glycerol, 0.2 mg ml^-1^ BSA, 1 mg ml^-1^ glucose oxidase, 0.04 mg ml^-1^ catalase, 0.8% dextrose and saturated Trolox (>5 mM)) (20). Cas9-RNA in the Cas9–RNA activity and imaging buffer was added to the flow chamber at the concentrations much higher (e.g. 100 nM ) than the dissociation constant of Cas9–RNA–DNA (18) for Cas9–RNA targeting of DNA and Cas9–RNA induced DNA unwinding. All the imaging experiments were done at room temperature and time resolution was either 100 or 35 ms per frame. The total fluorescence from each of the immobilized DNA targets molecules was optically split into two separate donor and acceptor optical paths. The emissions belonging to these parts were projected onto two halves of a cryo-cooled (<−70°C) EMCCD camera (Andor) which was stored as a video recording by the camera. The video recording containing fluorescent spots was then analyzed using custom scripts to extract background corrected donor fluorescence (*I*_D_), acceptor fluorescence (*I*_A_). FRET efficiency (*E*) of each detected spot was approximated as *E* = *I*_A_/(*I*_D_ + *I*_A_). In the analysis of DNA unwinding experiments, the DNA molecules with the missing or inactive acceptor label were avoided by only including the fluorescent spots in the acceptor channel. Data acquisition and analysis software are available at https://cplc.illinois.edu/software/. Most of the primary analysis was carried out using custom-written scripts. Methods describing the acquisition of smFRET data and analysis have been reported previously (24).

### *E* histograms and analysis of Cas9-RNA induced DNA unwinding and rewinding

For every single molecule, the first five data points of its *E* time-traces were used as data points to construct *E* histograms. More than 2000 molecules contributed to each *E* histogram. The donor only peak (*E* = 0), low FRET (0.2 < *E* < 0.6 or 0.65 or 0.70) and high FRET (*E* > 0.6 or 0.65 or 0.7) are three characteristic populations observed in these *E* histograms. Based on this low and high FRET populations, Cas9–RNA induced DNA unwinding was modeled as a two-state system, as shown below. The unwound fraction (*f*_unwound_) was calculated as a fraction of the low FRET population in the *E* histograms of DNA unwinding experiments.

$$\begin{equation*}\begin{array}{@{}l@{}} \mathop {{\rm{Cas}}9\hbox{-}{\rm{RNA\hbox{-}DNA}}}\limits_{\begin{array}{@{}*{1}{c}@{}} {\left( {{\rm{rewound}}} \right)}\\ {\left( {{\rm{high\ FRET}}} \right)} \end{array}} \rightleftharpoons \mathop {{\rm{Cas9\hbox{-}RNA\hbox{-}DNA}}}\limits_{\begin{array}{@{}*{1}{c}@{}} {\left( {{\rm{unwound}}} \right)}\\ {\left( {{\rm{low\ FRET}}} \right)} \end{array}} \mathop \to \limits^{{k_{{\rm{c,int}}}}} \mathop {{\rm{Cas9\hbox{-}RNA\hbox{-}DNA}}}\limits_{\left( {{\rm{cleaved}}} \right)} .\\ {k_{{\rm{c,}}{\mathop{\rm int}} }}\;{\rm{is\ the\ intrinsic\ rate\ of\ cleavage}} \end{array}\end{equation*}$$

### Calculation of *S*

*S* was calculated as the ratio of ${f_{unwound}}$for cognate DNA and aggregate of ${f_{unwound}}$ for DNA with PAM-distal mismatches, weighted by their ${n_{PD}}$.

$$\begin{equation*}S = \frac{{{f_{unwound}}\left( 0 \right)}}{{\mathop \sum \nolimits_{i = 1}^{{n_{PD}}} {n_{PD}} \times {f_{unwound}}\left( {{n_{PD}}} \right)\ }}\end{equation*}$$

${n_{PD}}$ is the number of PAM-distal mismatches, ${f_{unwound}}( 0 )$ is the unwound fraction for cognate DNA (${n_{PD}} = 0)$, and ${f_{unwound}}( {{n_{PD}}} )$ is the unwound fraction for DNA with a given ${n_{PD}}$.

## RESULTS

We previously developed an smFRET assay to investigate DNA unwinding in the PAM-distal region of the protospacer (23,24). Each DNA molecule was labeled with a donor (Cy3) and an acceptor (Cy5) fluorophore on the target and non-target strand, respectively. Dual labeled DNA targets were immobilized on a polymer-passivated quartz slide through biotin-neutravidin interaction and imaged using total internal reflection (TIRF) microscopy. Given the relatively short distance (9 bp) between the fluorophores, before adding Cas9–gRNA the FRET efficiency value (*E*) is high, ∼0.75. After addition of Cas9–gRNA and subsequent DNA binding and unwinding, the distance increases, lowering *E* values (Figure 1B and Supplementary Figure S2A). For these studies, we used catalytically inactive versions of Cas9 (referred to as dCas9s) to avoid potential complications arising from DNA cleavage and will omit the prefix ‘d’ here. We have previously shown that cleavage occurs from the unwound state, therefore the degree of DNA unwinding by the ‘nuclease-dead’ version likely reflects Cas9 target specificity (18). We acknowledge that there are differences in the post cleavage state conformations between dCas9 and nuclease active Cas9, but this is outside the scope of our study (9).

Mismatched DNA targets are denoted by their number of PAM distal mismatches relative to the gRNA (*n*_PD_). For example, *n*_PD_ = 2 refers to DNA with the 19th and 20^th^ base pairs (counting from PAM) mismatched relative to the gRNA. We showed previously that for WT Cas9, *E* for cognate DNA (the number of PAM distal mismatches = 0) is reduced from 0.75 to 0.3 upon Cas9-induced DNA unwinding (18). As more PAM-distal mismatches are introduced from 1 to 4, the low FRET unwound state is progressively converted to high FRET rewound state (18). The rewound state has the same *E* value as the DNA only (Figure 1C) but it is not due to dissocation of Cas9-gRNA ^18^. The exact number of bp unwound in the rewound state cannot be determined through *E* values but must be 9 bp or higher because binding is still very stable (18). Further, we showed that the relative fraction of the unwound state, *f*_unwound_, generally decreases with increasing PAM distal mismatches. This parallels the reduced target cleavage efficiency with increasing PAM distal mismatches. The fraction unwound versus the number of PAM distal mismatches curves showed steeper drops for eCas9 and Cas9–HF1 compared to WT Cas9 (Figure 1D), reflecting the enhanced sensitivity to PAM-distal mismatches which we previously proposed as one of the mechanisms of their improved cleavage specificity (18).

### HypaCas9 and SniperCas9 with canonical gRNA

For this current study, we used the smFRET assay to examine the sensitivity of newer EngCas9s, HypaCas9 and SniperCas9, to PAM distal mismatches. These studies were first performed with the canonical gRNA so that they could be directly compared to the first generation EngCas9s (eCas9 and Cas9-HF1). Interestingly, DNA fraction unwound for SniperCas9 was less sensitive to PAM distal mismatches than other variants although SniperCas9 was still more sensitive than WT Cas9. This suggests that the unwinding activity is more promiscuous for SniperCas9 compared to HypaCas9, eCas9 or Cas9–HF1 (Figure 1C and D).

**Figure 2.**
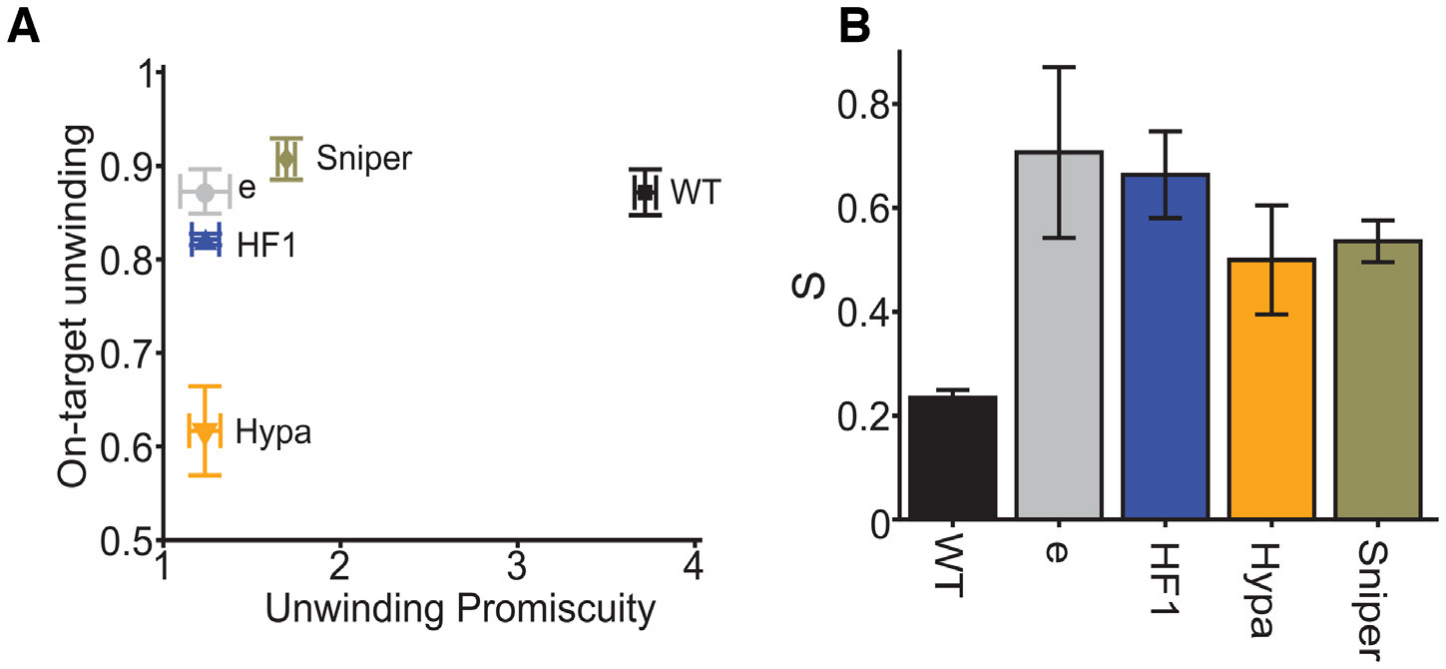
Unwinding specificity of different Cas9s with canonical gRNA. (**A**) A plot of on-target unwinding activity (*f*_unwound_ of cognate DNA) versus unwinding promiscuity for different Cas9s. (**B**) Unwinding specificity (*S*_unwinding_) for different Cas9s. Error bars represent SD. Data for dCas9, deCas9, and dCas9–HF1 is taken from a previous study (31).

For quantitative comparison of unwinding promiscuity across different variants of Cas9 and gRNA, we defined unwinding promiscuity as the sum of the number of PAM distal mismatches multiplied by the corresponding fraction unwound for DNA targets with one, two and three mismatches. In this definition, unwinding of DNA targets with more mismatches resulted in a higher penalty and consequently a higher promiscuity score. Figure 2A shows on-target unwinding activity (fraction unwound of DNA target with no mismatches) versus unwinding promiscuity for all five Cas9 variants paired with canonical gRNA. All EngCas9s have reduced unwinding promiscuity compared to WT Cas9. Except for HypaCas9, their on-target unwinding activities are not dramatically impaired compared to WT Cas9 (Figure 2A).

To further simplify this comparison, we defined a single value for specificity, *S*, by taking the ratio between on-target unwinding activity and unwinding promiscuity. The ideal system would have high on-target activity and low promiscuity resulting in a high specificity value. When paired with canonical gRNA the specificity for WT and EngCas9s were as follows: 0.23 for WT Cas9, 0.50 for HypaCas9, 0.53 for SniperCas9, 0.66 for Cas9–HF1 and 0.71 for eCas9 (Figure 2B). Specificity is low for WT Cas9 due to its high unwinding promiscuity. EngCas9s have higher specificity than WT Cas9, mainly because of reduced unwinding promiscuity. HypaCas9 has lower specificity than other EngCas9s due to its reduced on-target unwinding activity. In contrast, SniperCas9 has higher on-target unwinding activity but is more promiscuous compared to other EngCas9s.

### Extended gRNA

**Figure 3.**
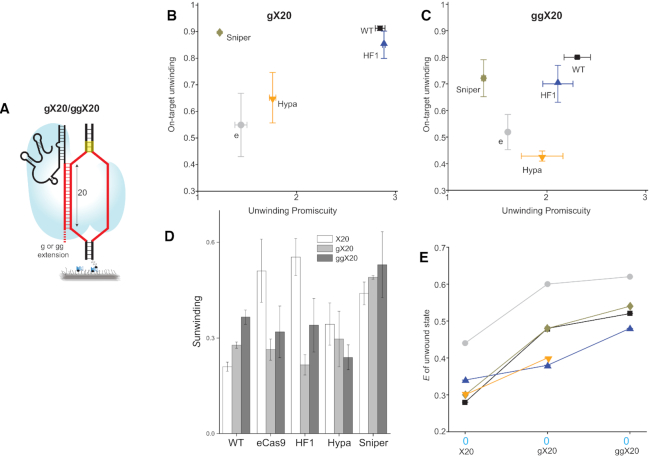
Unwinding specificity of extended gRNAs and the *E* values of their unwound state. (**A**) Schematic and naming convention of extended gRNA used in the unwinding assay. gX20 or ggX20 are gRNA with 5′g and 5′ gg non-hybridizing extensions respectively with canonical lengths of 20 bp in target strand hybridizing region. (**B**, **C**) A plot of on-target unwinding activity vs. unwinding promiscuity with gX20 (B) and ggX20 (C). (**D**) *S* compared between canonical and extended gRNAs. (**E**) *E* values of the unwound state compared between canonical (X20) and extended gRNAs. Error bars represent SD.

Next, we examined whether a system with higher specificity can be created by further altering the unwinding activity of Cas9s using non-canonical gRNAs. The canonical gRNA has 20 nucleotides (referred to here as X20) that are complementary to the protospacer. It has been shown that adding extra nucleotides at the 5′ end of X20 alters Cas9's activity (21,22). We wondered if Cas9 variants might have higher unwinding specificity with extended gRNAs. Therefore, we used the smFRET assay to test the DNA unwinding activity of WT and all four EngCas9 variants paired with two extended gRNAs. These two extended gRNAs are referred to here as gX20 and ggX20, where g and gg represent extra non-hybridizing guanines on the 5′ end of the RNA (Figure 3A and Supplementary Figure S3A). T7 or U6 *in vitro* transcription is often used to generate gRNAs, and addition of one or two 5′ g's to the sequence can increase transcription efficiency (26). If gX20 or ggX20 does not reduce activity or specificity, it would expand the repertoire of sequences that can be targeted for genome engineering.

The unwound and rewound FRET states were also observed with gX20 and ggX20 (Supplementary Figure S4B and C), and fraction unwound values were calculated for perfectly matched DNA as well as for DNA with 1–3 PAM distal mismatches as above. (Supplementary Figure S4D and E). Most notably, SniperCas9 paired with gX20 had an increase in specificity (Figure 3B and D). We observed that this improvement can be explained by a decreased unwinding promiscuity coupled with a maintenance of on-target activity. Strikingly, SniperCas9 paired with gX20 had increased sensitivity (lower fraction unwound) to DNA target containing a single PAM distal mismatch, compared to SniperCas9 paired with the canonical gRNA (Supplementary Figure S4D and Figure 1D). SniperCas9 paired with ggX20, however, lowered specificity back to near wild type levels (Figure 3D). This resulted from a decrease in on-target activity, suggesting that overextending the gRNA could negatively impact SniperCas9 DNA unwinding (Figure 3C). Pairing other EngCas9s with gX20 either reduced on-target activity, which was true for eCas9 and HypaCas9, or increased unwinding promiscuity (Cas9-HF1) (Figure 3B). Overall this resulted in reduced specificity for all EngCas9s (except SniperCas9) paired with gX20 (Figure 3D). WT Cas9 paired with gX20 showed an increase in specificity, which is mainly due to a decrease in unwinding promiscuity (Figure 3B and D). This increase in specificity was greater when WT Cas9 was paired with ggX20, which also resulted because of a decrease in unwinding promiscuity. However, a slight decrease in on-target activity was observed.

We also found that FRET efficiency values of the unwound state increase, to varying degrees, when Cas9s are paired with gX20 and ggX20 (Figure 3E). This suggests that the extra guanines decrease the number of PAM distal protospacer base pairs unwound. Finally, we note that, with gX20, Cas9-HF1 and HypaCas9 showed higher unwinding than eCas9 for DNA targets with and without mismatches (Supplementary Figure S4D) as we will discuss further below.

### Truncated gRNA

**Figure 4.**
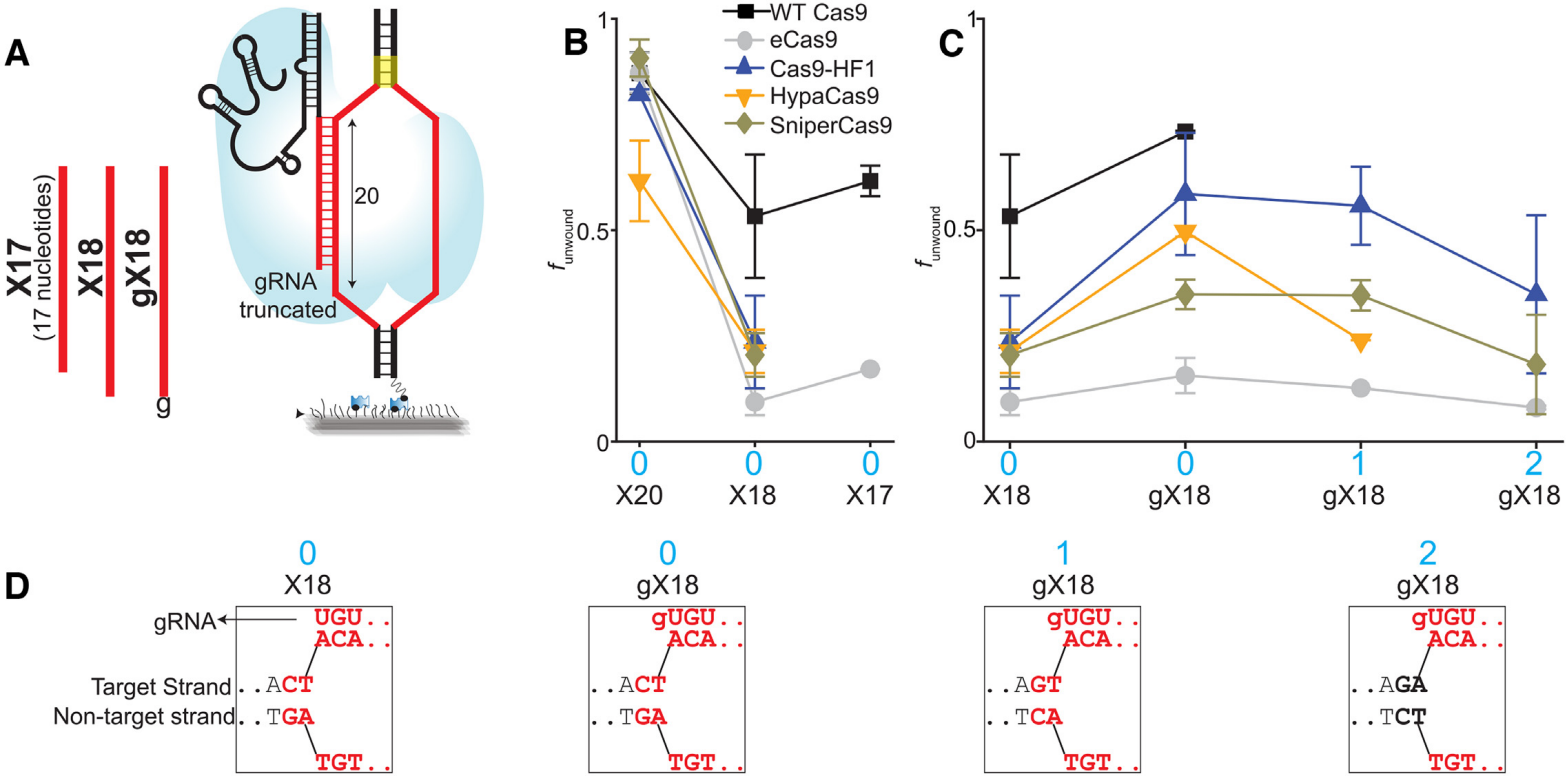
Unwinding by truncated gRNA. (**A**) Schematic and naming convention of truncated gRNA used in the unwinding assay. X18/X17 indicate the gRNA with length of 18/17 base pairs in the target strand hybridizing region, as opposed to the length of 20 bp for canonical gRNA. Addition of a mismatched g at the 5′ end of X18 gives gX18. (**B**) Comparison of *f*_unwound_ between X20, X18, and X17 for cognate DNA. (**C**) *f*_unwound_ with the different gRNA–Cas9 combinations for different DNA with 18 base pairs between gRNA and target strand. (**D**) The PAM-distal sequences of such gRNA and DNA combination are shown below for each column. All these DNA targets present the same number of 18 matching sequences to the gRNAs but differ in the sequence only beyond these 18 matching sequences at the PAM-distal site. Such differing nucleotides are marked in black. Data for dCas9, deCas9 and dCas9–HF1 with X20 is taken from a previous study (31). Error bars represent SD.

It has been shown that the truncation of gRNAs at the 5′ end (PAM-distal side) by two or three nucleotides, referred to here as X18 or X17, respectively, also alters Cas9's activity (Figure 4A and Supplementary Figure S3B) (20). We used our smFRET assay to examine the impact of pairing these truncated gRNAs with all five Cas9 variants on DNA unwinding activity. Similar to canonical and extended gRNAs, truncated gRNA showed both the unwound and rewound FRET states (Supplementary Figures S5 and S6A).

As mentioned previously, Cas9 uses the base pairing of 20 nucleotides between gRNA and protospacer to completely open the DNA molecule. Thus, it was not surprising that all Cas9s showed a decrease in fraction unwound when X18 or X17 was used in place of the canonical gRNA (Figure 4B). Note that Cas9s that showed abolished unwinding with X18 were not tested on other DNA targets or with X17 gRNA, because their fraction unwound values were likely to be below our detection limit. Surprisingly, adding an unmatched guanine to the 5′ end of X18 (referred to here as gX18) increased the fraction of unwound molecules when paired with WT Cas9 and all EngCas9s except eCas9 (Figure 4C and D and Supplementary Figure S6). gX18 is similar to having a 19 nucleotide gRNA with one PAM distal mismatch, in this case guanine. In addition, using gX18 against three DNA targets having the same 18 base pair matches between gRNA and protospacer, but different nucleotides in the 19th and 20th positions of the protospacer (counting from the PAM), showed different fraction unwound values (Supplementary Figure S6). Overall, the data suggest that unwinding depends on gRNA length, nucleotide composition, and protospacer sequence composition, especially in the PAM-distal region. This highlights the complexity of PAM distal sequence dependence in establishing the fully unwound state.

Cas9-HF1 and HypaCas9 consistently showed higher unwinding fraction than eCas9 when they are paired with gX18, similarly to when paired with gX20 (Figure 4C). This difference in unwinding activity between Cas9-HF1/HypaCas9 and eCas9 with both gX20 and gX18 is striking because no such difference was observed amongst them when paired with the canonical gRNA (Figure 1D, Supplementary Figure S4B and C and Figure 4C). Finally, we note that truncated gRNAs cannot base pair with the full 20 nucleotides of the protospacer, hence unwinding promiscuity or specificity values, as we defined in this work, cannot be determined.

## DISCUSSION

Here, we presented a summary of our systematic analysis of DNA unwinding specificity, using different combinations of EngCas9s and non-canonical gRNAs. This approach allowed us to make a quantitative comparison of unwinding specificity between different combinations. We were also able to examine the relationship between on-target unwinding activity and unwinding promiscuity. Notably HypaCas9 and SniperCas9, newer EngCas9s, had lower specificity of unwinding than the first generation EngCas9s, eCas9 and Cas9–HF1 when paired with the canonical gRNA. Using canonical gRNA, specificity was in the following increasing order, WT, Hypa, Sniper, HF1 and eCas9. In order to rationalize this observation, we turn to the available structural data. HypaCas9 mutations were introduced into the REC3 domain. This domain is responsible for recognizing the unwound state and transmitting the signal to the HNH domain for nuclease activation (8,12,18). The reduced unwinding of the cognate DNA target for HypaCas9 suggests that contacts between the target strand and the polar residues of REC3 stabilize the unwound state. Despite a more rational approach behind the design of HypaCas9(12), its on/off target ratio in cellular cleavage efficiency is not very different from eCas9/Cas9–HF1 (15). Our observation of low on-target activity may explain the in cell results.

We also found that combining WT Cas9 with extended gRNAs (gX20 and ggX20) improves unwinding specificity by reducing unwinding promiscuity. However, extended gRNAs reduce unwinding specificity for all EngCas9s except SniperCas9, by either reducing the on-target activity (Hypa, eCas9) or increasing the unwinding promiscuity (HF1). SniperCas9, however, benefited from being combined with extended gRNA by reducing the unwinding promiscuity in both cases (gX20 & ggX20). The combination of gX20 and SniperCas9 had the highest unwinding specificity of all combinations tested.

We noted that the FRET efficiency of the unwound state increased as the gRNA length increased. We can again lean on structural data to help explain this phenomenon. Structures of Cas9-gRNA-DNA with canonical gRNA show that the RuvC domain of Cas9 makes direct contacts with the 5′ end of the gRNA (27) (Supplementary Figure S7A), effectively capping its 5′ end. Therefore, accommodation of extensions on the 5′ end of the gRNA is likely to cause distortion of the gRNA-DNA as seen in the structure of Cas9-gRNA-DNA with ggX20 (Supplementary Figure S7A) (28). This may impact the stability of the unwound state. Our observation that FRET efficiency values of the unwound state increase with gRNA extension is consistent with such structural distortion.

WT Cas9 paired with truncated gRNAs showed reduced unwinding overall, which may underlie the cleavage specificity improvements observed in cells. Having a lower unwinding for off targets would be beneficial for accuracy. However, the reduction in on-target unwinding activity would be detrimental to situations where a high cleavage efficiency is desired. A previous study used smFRET analysis of doubly labeled WT Cas9 to monitor the movement of HNH domain between inactive and active states. They showed that HNH activation decreases when increasing amount of PAM distal mismatches are introduced, and when gRNA is truncated (29). This parallels our observation of a fraction unwound decrease with increasing PAM distal mismatches and gRNA truncation. For WT Cas9 with X20, HNH activation is 78–100 % of fraction unwound for all DNA targets, indicating that most unwound DNA states lead to HNH activation (Supplementary Figure S5C) (29). When using X17 gRNA, HNH activation was only 45% of fraction unwound (29), indicating more than half of unwound states do not generate HNH activation with X17 (Supplementary Figure S5C). gRNA truncation may make it difficult for REC3 to recognize the RNA–DNA hybrid and transmit DNA unwinding towards downstream HNH activation because REC3 is responsible for sensing the formation of RNA–DNA hybrid via its interactions with both target strand and gRNA (27,30).

Overall, EngCas9s had reduced on-target unwinding activity with truncated gRNAs. However, an increase in DNA unwinding was observed when a single unmatched guanine was added to the 5′ end of X18 for some combinations. Interestingly, when the DNA targets were changed such that nucleotides outside of the gRNA base pairing region were different, the fraction unwound values also varied. The unmatched guanine of gX18 may form interactions with the DNA target, as observed in the structure of Cas9–ggX20–DNA (32) (Supplementary Figure S7B). Such interactions may potentially influence unwinding activity in a PAM-distal DNA sequence dependent manner. Cas9–HF1 and HypaCas9 reduce unwinding through mutations to residues nearby the PAM distal end. We speculate that repositioning of the RNA-DNA hybrid duplex induced by the extra guanine partially relieves DNA unwinding defects of Cas9–HF1/HypaCas9 mutations. This partial relief leads to an increase in Cas9–HF1/HypaCas9's unwinding compared to eCas9, whose mutations affect non-target strand and not the RNA–DNA hybrid.

The non-canonical gRNAs can expand genome engineering applications in the following ways. First, efficient T7 or U6 transcription of gRNA require 5′g or 5′gg (26,36). Due to this requirement, only the gRNA with 5′ g in its target strand hybridizing region are routinely employed. This restricts the number of available gRNA target sites (37). However, the 5′g and 5′gg extensions in the gRNA not only satisfy this requirement to expand potential target sites, but also provides improved specificity for wild type Cas9 and SniperCas9. Second, transfection of non-canonical gRNA may provide an alternative path toward obtaining higher specificity in existing cell and animal systems that express WT Cas9. Third, the ultrastability of Cas9–RNA–DNA even after cleavage may prevent the exposure of the cleaved site until motor proteins such as RNA polymerases can help remove Cas9–RNA from the DNA (38). Weakening the Cas9–RNA–DNA complex by EngCas9 mutations (39) or non-canonical gRNA may facilitate easier Cas9–RNA removal and gene editing. Therefore, an appropriate combination of Cas9 and/or non-canonical gRNA may provide not only an improved on-target cleavage activity and specificity, but also higher gene editing efficiencies.

The types of non-canonical gRNA are also continually expanding, including gRNA with DNA nucleotides (33), chemical modifications (34,35) whose specificity improvement may also arise from DNA unwinding and cleavage activation becoming more sensitive to mismatches. Furthermore, many Cas9 variants are also being developed and can be studied using this assay. One limitation of our current assay is that we only can detect unwinding in the PAM distal region, therefore we focused on PAM distal mismatches. However, with judicious placement of donor and acceptor fluorophores, and various DNA targets and mismatch types, it will become possible to study the mismatch effects on unwinding of other parts of the DNA

## Supplementary Material

gkz1058_Supplemental_File

## Notes

Present address: Digvijay Singh, Division of Biological Sciences, University of California San Diego, La Jolla, CA 92095-0377, USA.
