# Supplementary material for: Single molecule analysis of effects of non-canonical guide RNAs and specificity-enhancing mutations on Cas9-induced DNA unwinding
Source: Nucleic Acids Res. 2019 Nov 12;47(22):11880–8. doi: 10.1093/nar/gkz1058 (PMC7145707; doi:10.1093/nar/gkz1058)
Supplement: gkz1058_Supplemental_File [file gkz1058_supplemental_file.pdf]

Supplementary Information for

Single molecule analysis of effects of non-canonical guide RNAs and specificity-enhancing mutations on Cas9-induced DNA unwinding

Ikenna C. Okafor[1, *], Digvijay Singh[2, #, *], Yanbo Wang[2, *], Minhee Jung[3], Haobo Wang[4], John Mallon[4], Scott Bailey[2,4], Jungjoon K. Lee[3] and Taekjip Ha[2, 5-7,@]

[1]Department of Biology, Johns Hopkins University, Baltimore, Maryland 21218, USA

[2]Department of Biophysics and Biophysical Chemistry, Johns Hopkins University School of Medicine, Baltimore, Maryland 21205, USA.

[3]Toolgen, Seoul 08501, Republic of Korea.

[4]Bloomberg School of Public Health, Johns Hopkins University School of Medicine, Baltimore, Maryland 21205, USA.

[5]Department of Biophysics, Johns Hopkins University, Baltimore, MD 21218, USA.

[6]Department of Biomedical Engineering, Johns Hopkins University, Baltimore, Maryland 21205, USA.

[7]Howard Hughes Medical Institute, Baltimore, Maryland 21205, USA.

[#]Present address: Division of Biological Sciences, University of California San Diego, La Jolla, California, 7 USA, 92095-0377.

[@]To whom correspondence should be addressed: Taekjip Ha (tjha@jhu.edu)

[*]These authors are co-first authors.

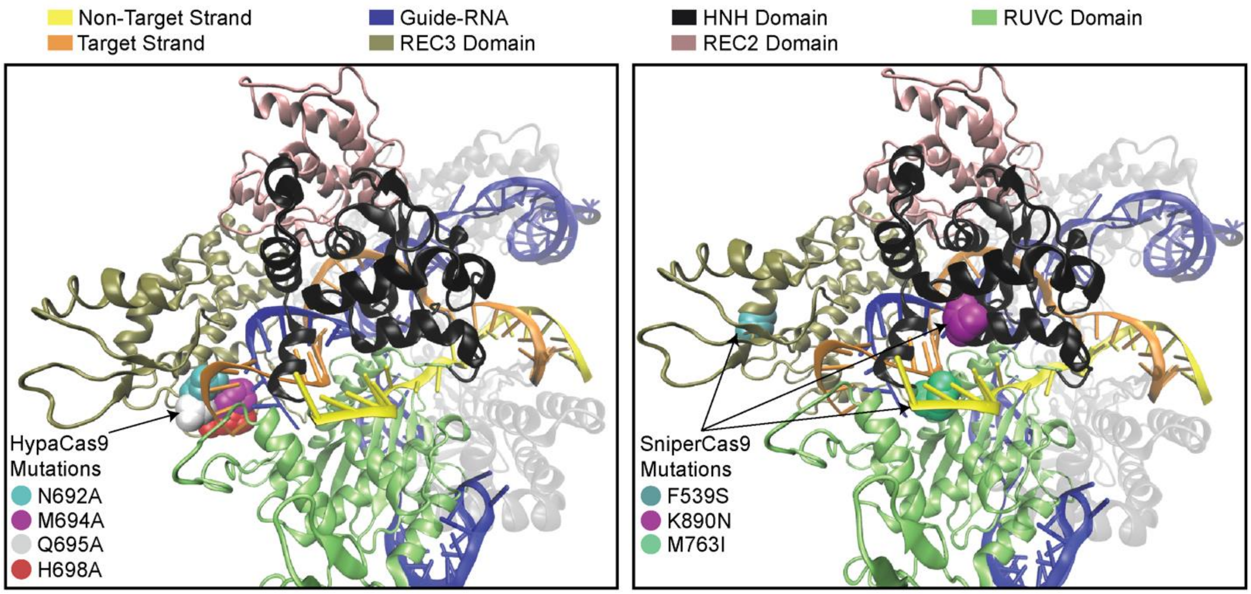

Figure S1. Locations of HypaCas9 and SniperCas9 mutations in the Cas9-RNA-DNA. (PDB ID: 5F9R(1)).

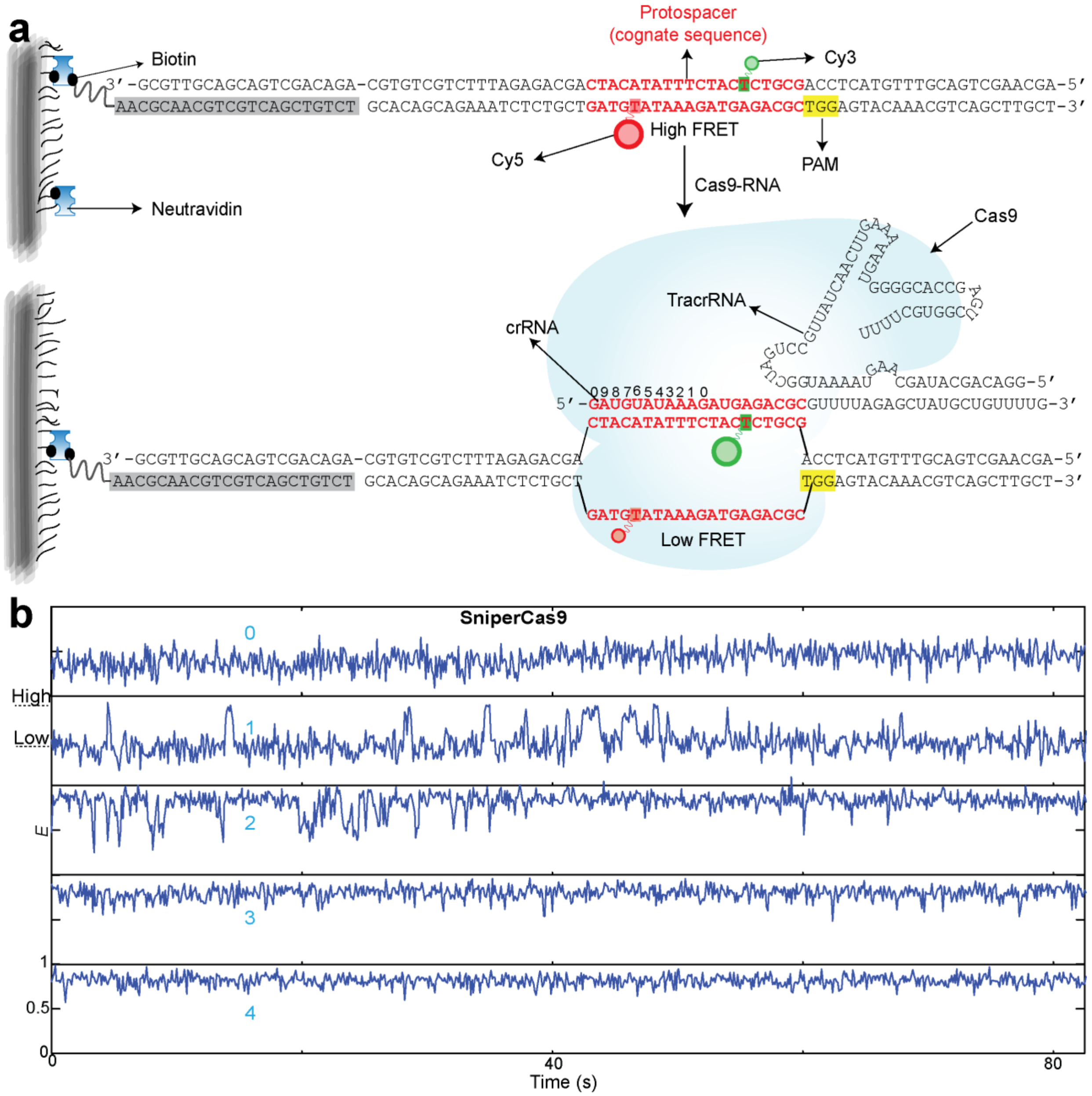

Figure S2. Locations of FRET probes for smFRET DNA unwinding experiments and *E* time-traces of DNA unwinding by SniperCas9-X20. (a) Schematic of Cas9-RNA-DNA complex. crRNA hybridized with tracrRNA is the guide-RNA, and the denoted red sequences hybridize with one of the strands (target strand) in the DNA. The strand complementary to target strand in the DNA is the non-target strand which also contains the PAM (5′-NGG-3′) as indicated. Highlighted in grey is a 22 nt biotin-labeled strand which is used as an adaptor for surface immobilization of the DNA. The donor and acceptor labels are conjugated to the 6th nucleotide (from PAM) in target strand and 16th in the non-target strand. Cas9-RNA binds the DNA leading to the unwinding of the DNA which results in an increase in distance between FRET labels and thus a

decrease in *E*. **(b)** Representative *E* time-traces of DNA unwinding by SniperCas9-X20 for different DNA targets.

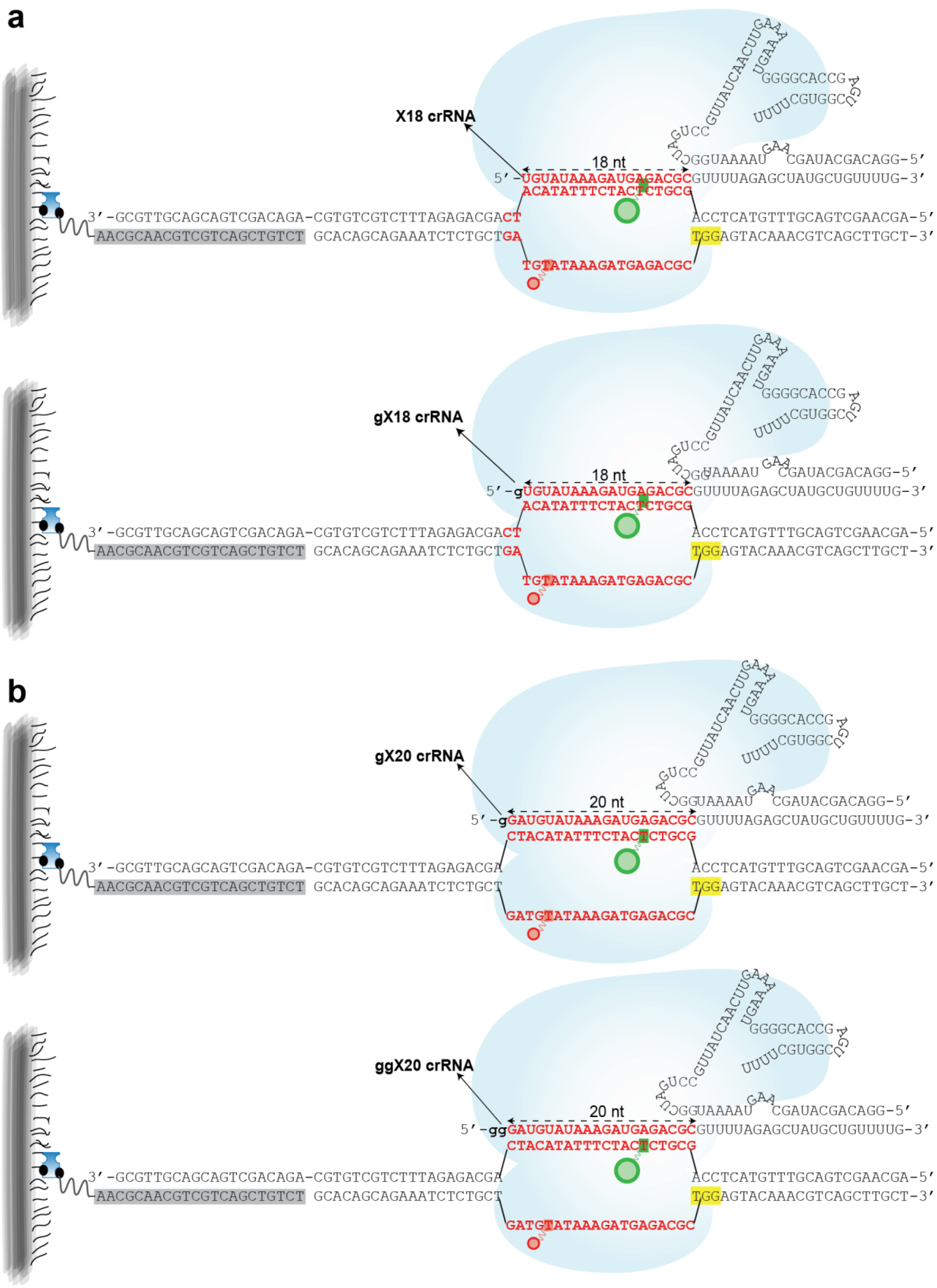
a
X18 crRNA
18 nt
5′-UGUAUAAAGAUGAGACGCGUUUUAGAGCUAUGCUGUUUUG-3′
CGAUACGACAGG-5′
ACATATTTCTACTCTGCG
3′-GCGTTGCAGCAGTCGACAGA-CGTGTCGTCTTTAGAGACGACT
AACGCAACGTCGTCAGCTGTCT GCACAGCAGAAATCTCTGCTGA
ACCTCATGTTTGCAGTCGAACGA-5′
TGGAGTACAAACGTCAGCTTGCT-3′
TGTATAAAGATGAGACGC
gX18 crRNA
18 nt
5′-gUGUAUAAAGAUGAGACGCGUUUUAGAGCUAUGCUGUUUUG-3′
CGAUACGACAGG-5′
ACATATTTCTACTCTGCG
3′-GCGTTGCAGCAGTCGACAGA-CGTGTCGTCTTTAGAGACGACT
AACGCAACGTCGTCAGCTGTCT GCACAGCAGAAATCTCTGCTGA
ACCTCATGTTTGCAGTCGAACGA-5′
TGGAGTACAAACGTCAGCTTGCT-3′
TGTATAAAGATGAGACGC
b
gX20 crRNA
20 nt
5′-gGAUGUAUAAAGAUGAGACGCGUUUUAGAGCUAUGCUGUUUUG-3′
CGAUACGACAGG-5′
CTACATATTTCTACTCTGCG
3′-GCGTTGCAGCAGTCGACAGA-CGTGTCGTCTTTAGAGACGA
AACGCAACGTCGTCAGCTGTCT GCACAGCAGAAATCTCTGCT
ACCTCATGTTTGCAGTCGAACGA-5′
TGGAGTACAAACGTCAGCTTGCT-3′
GATGTATAAAGATGAGACGC
ggX20 crRNA
20 nt
5′-ggGAUGUAUAAAGAUGAGACGCGUUUUAGAGCUAUGCUGUUUUG-3′
CGAUACGACAGG-5′
CTACATATTTCTACTCTGCG
3′-GCGTTGCAGCAGTCGACAGA-CGTGTCGTCTTTAGAGACGA
AACGCAACGTCGTCAGCTGTCT GCACAGCAGAAATCTCTGCT
ACCTCATGTTTGCAGTCGAACGA-5′
TGGAGTACAAACGTCAGCTTGCT-3′
GATGTATAAAGATGAGACGC

Figure S3. Schematic of the smFRET unwinding assay involving non-canonical gRNAs. (a) Extended gRNAs. (b) Truncated gRNAs.

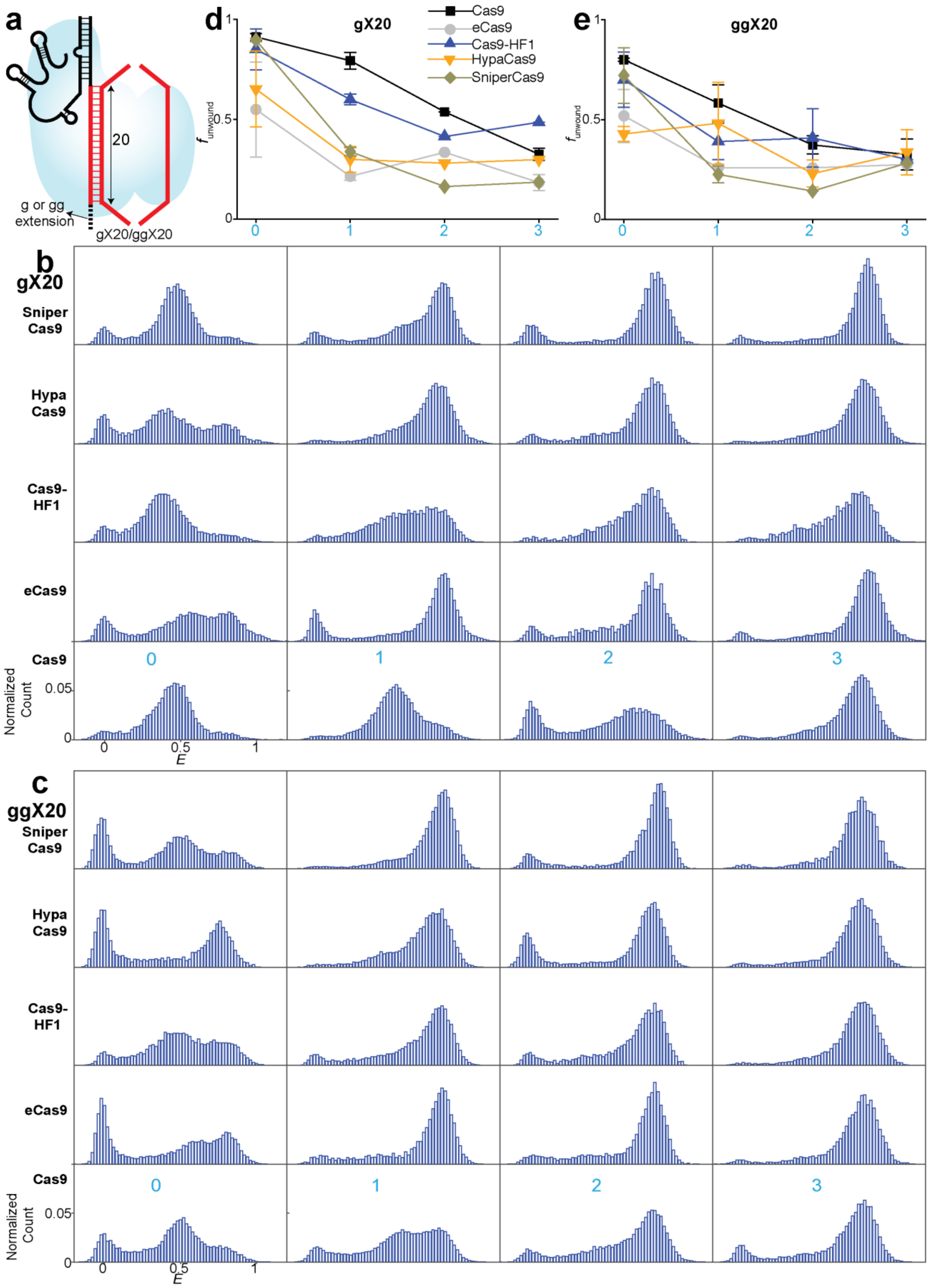

a
g or gg extension
gX20/ggX20
20
d
gX20
e
ggX20
Cas9
eCas9
Cas9-HF1
HypaCas9
SniperCas9
$f_{unwound}$
0
0.5
1
0
1
2
3
b
gX20
Sniper Cas9
Hypa Cas9
Cas9-HF1
eCas9
Cas9
Normalized Count
0.05
E
c
ggX20

Figure S4. Unwinding by extended gRNA.

(a) Schematic and naming convention of extended gRNA used in the unwinding assay. gX20 or ggX20 are gRNA with 5'g and 5' gg non-hybridizing extensions respectively with canonical lengths of 20 bp in target strand hybridizing region. **(b-c)** *E* histograms of unwinding by extended gRNA. Each row corresponds to a particular labeled Cas9. Each column corresponds to a particular DNA target. **(b)** Histograms by gX20. **(c)** Histograms by ggX20. **(d)** f_{unwound} vs. n_{PD} for different Cas9s with gX20. **(e)** f_{unwound} vs. n_{PD} for different Cas9s with ggX20.

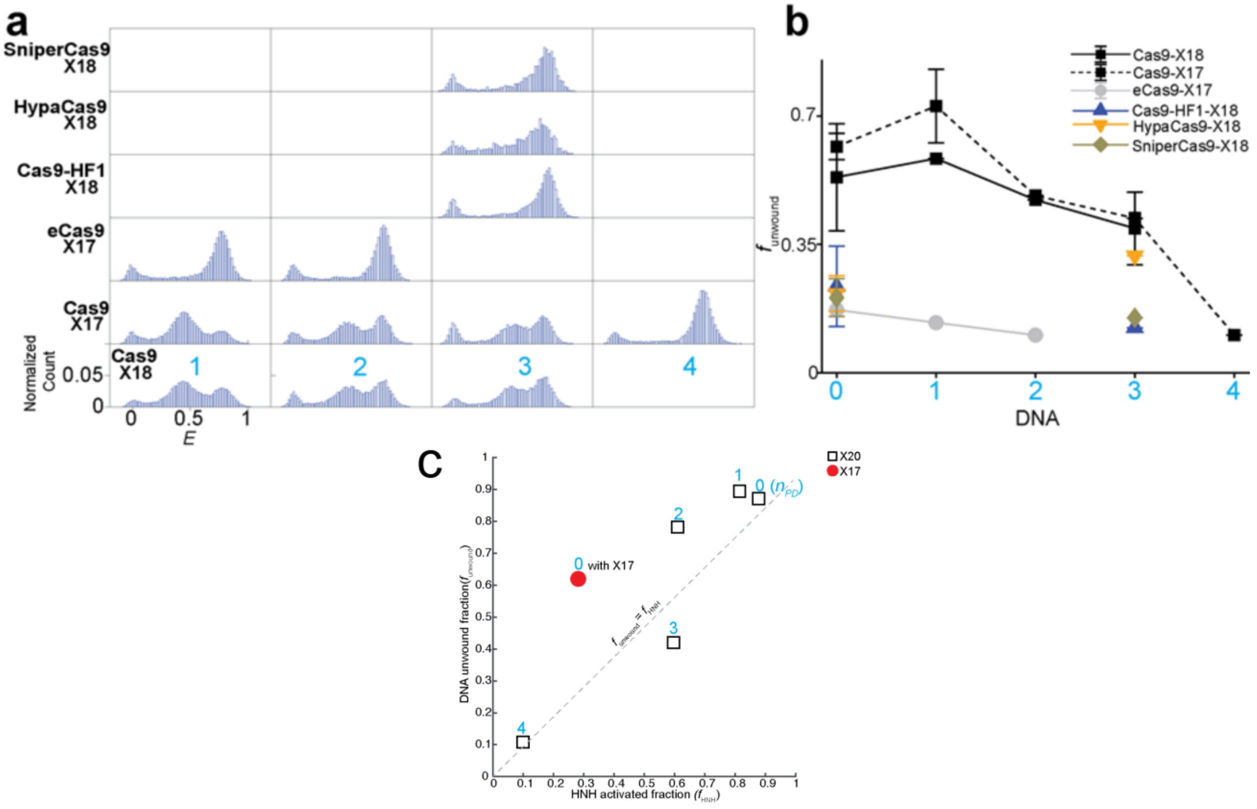

Figure S5. Unwinding by truncated gRNA.

(a) *E* histograms of unwinding by truncated gRNA. Each row corresponds to a particular labeled Cas9 and gRNA combination. Each column corresponds to a particular DNA target. **(b)** f_{unwound} vs. n_{PD} for different Cas9s with truncated gRNAs. **(c)** f_{unwound} vs. f_{HNH} activation for X20 and X17 (Red) for DNA targets with different n_{PD}

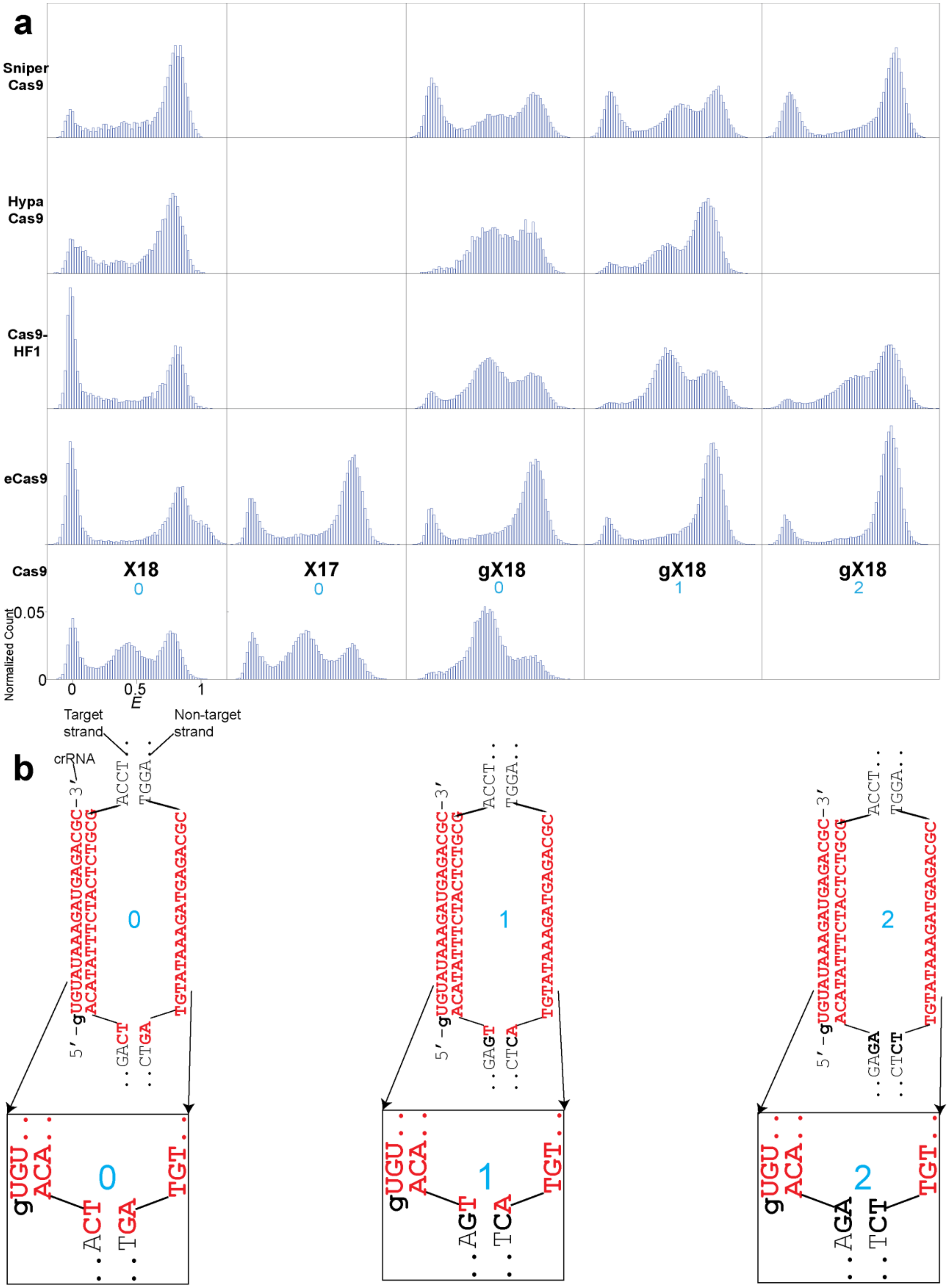
a
Sniper Cas9
Hypa Cas9
Cas9-HF1
eCas9
Cas9
X18
0
X17
0
gX18
0
gX18
1
gX18
2
Normalized Count
0.05
0
0
0.5
1
E
Target strand
Non-target strand
b
crRNA
5′-gUGUAUAAAGAUGAGACGC-3′
ACATATTTCTACTCTGCG
TGTATAAAGATGAGACGC
ACCT
TGGA
0
1
2

Figure S6. Unwinding by truncated gRNA.

(a) *E* histograms of unwinding by truncated gRNA. Each row corresponds to a particular labeled Cas9. Each column corresponds to a particular gRNA and DNA target. **(b)** Sequences of DNA and crRNA (of truncated gRNAs). X18/gX18 guide RNA forms the same Watson-Crick base pairing with n_{PD}=0, n_{PD}=1, and n_{PD}=2 DNA targets.

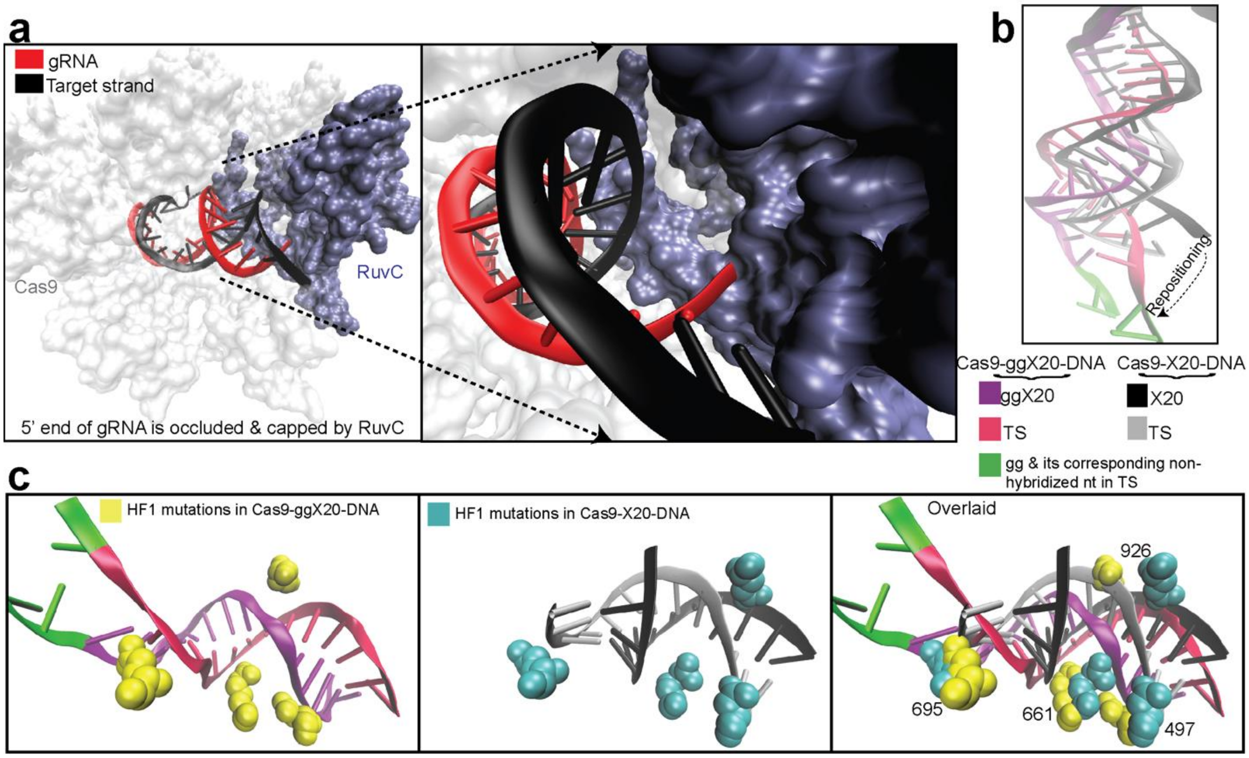

Figure S7. PAM distal end of Cas9-RNA-DNA complex.

(a) 5' end of the gRNA is occluded and capped by the RuvC nuclease domain in Cas9-X20-DNA. PDB ID: 5F9R(1). **(b)** The gRNA and target strand of Cas9-ggX20-DNA and Cas9-X20-DNA after aligning the residues of Cas9 between the two structures (Cas9-ggX20-DNA; PDB ID: 5Y36(2) and Cas9-X20-DNA; PDB ID: 5F9R(1)). With the extensions, the RNA-DNA hybrid at the PAM-distal end has been considerably repositioned, compared with its position with the canonical gRNA. The 21st and 22nd nucleotide, counting from PAM, of the target strand are not base-paired with the non-target strand nucleotides, but are rather flipped towards the gg of the ggX20. **(c)** The relative changes in positions of RNA-DNA hybrid and Cas9-HF1 mutations between Cas9-X20-DNA and Cas9-ggX20-DNA.

Table S1: gRNA and DNA targets used for Cas9-RNA induced DNA unwinding smFRET assay.

DNA	Sequences
0/0	20 nucleotide biotinylated adaptor for surface immobilization<br>5′-*-AACGCAACGTCGTCAGCTGTCT GCACAGCAGAAATCTCTGCTGATGTATAAAGATGAGACGCTGGAGTACAAACGTCAGCTTGCT-3′<br>3′-GCGTTGCAGCAGTCGACAGA-CGTGTCGTCTTTAGAGACGACTACATATTTCTACTCTGCGACCTCATGTTTGCAGTCGAACGA-5′
1	5′-*-AACGCAACGTCGTCAGCTGTCT GCACAGCAGAAATCTCTGCTCATGTATAAAGATGAGACGCTGGAGTACAAACGTCAGCTTGCT-3′<br>3′-GCGTTGCAGCAGTCGACAGA-CGTGTCGTCTTTAGAGACGAGTACATATTTCTACTCTGCGACCTCATGTTTGCAGTCGAACGA-5′
2	5′-*-AACGCAACGTCGTCAGCTGTCT GCACAGCAGAAATCTCTGCTCTTGTATAAAGATGAGACGCTGGAGTACAAACGTCAGCTTGCT-3′<br>3′-GCGTTGCAGCAGTCGACAGA-CGTGTCGTCTTTAGAGACGAGAACATATTTCTACTCTGCGACCTCATGTTTGCAGTCGAACGA-5′
3	5′-*-AACGCAACGTCGTCAGCTGTCT GCACAGCAGAAATCTCTGCTCTAGTATAAAGATGAGACGCTGGAGTACAAACGTCAGCTTGCT-3′<br>3′-GCGTTGCAGCAGTCGACAGA-CGTGTCGTCTTTAGAGACGAGATCATATTTCTACTCTGCGACCTCATGTTTGCAGTCGAACGA-5′
4	5′-*-AACGCAACGTCGTCAGCTGTCT GCACAGCAGAAATCTCTGCTCTACTATAAAGATGAGACGCTGGAGTACAAACGTCAGCTTGCT-3′<br>3′-GCGTTGCAGCAGTCGACAGA-CGTGTCGTCTTTAGAGACGAGATGATATTTCTACTCTGCGACCTCATGTTTGCAGTCGAACGA-5′
**RNA**	**Sequences**
Canonical crRNA (X20)	5′-GAUGUAUAAAGAUGAGACGCGUUUUAGAGCUAUGCUGUUUUG-3′
X17 crRNA	5′-GUAUAAAGAUGAGACGCGUUUUAGAGCUAUGCUGUUUUG-3′
X18 crRNA	5′-UGUAUAAAGAUGAGACGCGUUUUAGAGCUAUGCUGUUUUG-3′
gX20 crRNA	5′-gGAUGUAUAAAGAUGAGACGCGUUUUAGAGCUAUGCUGUUUUG-3′
ggX20 crRNA	5′-ggGAUGUAUAAAGAUGAGACGCGUUUUAGAGCUAUGCUGUUUUG-3′
gX18 crRNA	5′-gUGUAUAAAGAUGAGACGCGUUUUAGAGCUAUGCUGUUUUG-3′
tracRNA	5′-GGACAGCAUAGCAAGUUAAAAUAAGGCUAGUCCGUUAUCAACUUGAAAAAGUGGCACCGAGUCGGUGCUUUUU-3′

Thymine modification for Cy3 and Cy5 labeling. *Biotin. PAM.DNA sequences complementary to guide RNA are shown in red (Cognate). RNA sequences complementary to the protospacer in a cognate DNA target are shown in red (Cognate).

REFERENCES

1. Jiang,F., Taylor,D.W., Chen,J.S., Kornfeld,J.E., Zhou,K., Thompson,A.J., Nogales,E. and Doudna,J.A. (2016) Structures of a CRISPR-Cas9 R-loop complex primed for DNA cleavage. *Science*, **351**, 867–71.
2. Huai,C., Li,G., Yao,R., Zhang,Y., Cao,M., Kong,L., Jia,C., Yuan,H., Chen,H., Lu,D., *et al.* (2017) Structural insights into DNA cleavage activation of CRISPR-Cas9 system. *Nat. Commun.*, **8**, 1375.
